# Hooked for Decay with Hydrophobic‐Coated Magnetic Beads to Grapple and Disintegrate Nanoplastics

**DOI:** 10.1002/anie.202510356

**Published:** 2025-08-19

**Authors:** Dong Wang, Maochao Mao, Maximilian Lorberg, Julian Luka, Marian Bienstein, Jun Okuda, Ulrich Schwaneberg

**Affiliations:** ^1^ Institute of Biotechnology RWTH Aachen University Worringerweg 3 52074 Aachen Germany; ^2^ Institute of Technical and Macromolecular Chemistry RWTH Aachen University Worringerweg 2 52074 Aachen Germany; ^3^ Institute of Inorganic Chemistry RWTH Aachen University Landoltweg 1 52074 Aachen Germany

**Keywords:** Biohybrid catalyst, Nanoplastics, SBR degradation, SPIONs, Water remediation

## Abstract

Degradation of synthetic polymers inevitably leads to the formation of nanoplastics (NPs), and recent studies associate health risks with NPs. Therefore, catching and degrading NPs are important to manage environmental and human health risks. In this study, we developed a biohybrid catalyst system with two functionalities to capture (Hook) and degrade (Decay) NPs. The biohybrid catalyst is composed of a Grubbs‐Hoveyda (GH) type cofactor conjugated to the material‐binding peptide LCI_F16C, which immobilizes the GH‐cofactor onto superparamagnetic iron oxide nanoparticles (SPIONs). The anchored biohybrid catalyst initiates a ring‐opening metathesis polymerization to generate a hydrophobic polynorbornene film around iron oxide core, which enables to efficiently capture and remove a broad spectrum of hydrophobic NPs, including polypropylene, polyethylene, polystyrene (PS), poly(ethylene terephthalate), poly(methyl methacrylate), and styrene‐butadiene rubber (SBR), with hydrophobic interaction as the main driving force. This hydrophobic coating facilitated rapid adsorption of PS‐COOH_500 nm_ NPs within 10 min, reaching an impressive adsorption capacity of 5.53 ± 0.29 g/g on PS NPs, and demonstrated a high recovery efficiency of 99% for SBR NPs. Notably, the embedded GH‐cofactor preserved its ethenolysis activity, cleaving internal C═C bonds of SBR and resulting in 6.5% degradation, thereby validating the concept of simultaneous nanoplastic capture and catalytic breakdown.

## Graphical abstract

Biohybrid catalysts based on the material‐binding peptide LCI_F16C, which is conjugated with a Grubbs‐Hoveyda cofactor, were immobilized on the Fe_3_O_4_ nanoparticles. The immobilized biohybrid catalysts catalyzed a ring‐opening metathesis polymerization (ROMP) on the surface of Fe_3_O_4_ nanoparticles to form a hydrophobic polynorbornene film. These film‐coated Fe_3_O_4_ nanoparticles exhibited bifunctional activity toward both capturing of nanoplastics and degrading of styrene‐butadiene rubber nanoparticles.

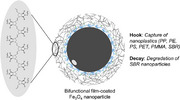

Plastic pollution has emerged as an important environmental concern, with micro‐ and nanoplastics (MP/NPs) posing particular challenges that require effective management strategies to ensure environmental and human health.^[^ ^1^, ^2^, ^3^, ^4^, ^5^, ^6^, ^7^ ^]^ MP/NPs‐free water systems are often challenging to obtain since conventional treatment methods such as coagulation, sedimentation, filtration, and disinfection are insufficient to effectively remove MP/NPs.^[^ ^8^, ^9^, ^10^ ^]^ Many common polyolefins, such as polyethylene, polystyrene, and non‐natural rubbers, are hardly biodegradable in nature due to their chemical inertness and lack of efficient biocatalysts.^[^ ^11^, ^12^, ^13^ ^]^ Elastomers, such as styrene butadiene rubber (SBR), play a significant role in environmental contamination. SBR, a main component of tire polymers, deteriorates as similar elastomers into micro‐/nanoscopic fragments through processes such as abrasion and weathering.^[^ ^14^ ^]^ SBR particles, ranging from nanometers to millimeters in size, are considered an important source of microplastic pollution.^[^ ^15^ ^]^ Tire‐related particles are released into the environment at rates of 0.2–5.5 kg per inhabitant per year, which raises concerns about their potential impact on ecosystems through their persistence.^[^ ^16^ ^]^

Recently, superparamagnetic iron oxide nanoparticles (SPIONs) have gained attention as adsorbent materials to enrich organic molecules from aqueous suspensions. SPIONs combine a highly active surface area with the ease of magnetic recovery from water and are inexpensively available in large quantities. For instance, unmodified SPIONs were reported to effectively remediate glyphosate from water, while specifically designed core‐shell or double‐shell SPIONs exhibited a high efficiency in extracting hydrocarbons and crude oil, as well as removing toxic organic compounds.^[^ ^17^, ^18^, ^19^, ^20^, ^21^, ^22^, ^23^ ^]^ The functionalization of SPIONs enabled adsorption of plastic nanoparticles via electrostatic and van der Waals interactions, which could be collected by exposure to an external magnetic field.^[^ ^24^, ^25^, ^26^, ^27^, ^28^, ^29^, ^30^, ^31^, ^32^, ^33^, ^34^ ^]^ In particular, the chemically modified SPION systems achieved maximum adsorption capacities of up to 1 g/g for MP/NPs and demonstrated reusability for at least three cycles.^[^ ^26^, ^29^, ^34^ ^]^

Material‐binding peptides (MBPs) are versatile peptides that interact with a variety of surfaces, including stainless steel^[^ ^35^, ^36^, ^37^ ^]^ and polymers^[^ ^38^ ^]^ through electrostatic, polar, hydrophobic, and hydrogen bond interactions.^[^ ^39^, ^40^, ^41^ ^]^ MBPs are compatible with standard and scalable application methods such as spraying or dip coating.^[^ ^42^ ^]^ A prominent example is the MBP LCI (liquid chromatography peak I, 47 amino acids) from *Bacillus subtilis*, which has been reported to bind to many polymer surfaces, such as polystyrene^[^ ^43^, ^44^ ^]^ and polypropylene^[^ ^42^, ^45^ ^]^ with high surface coverages. MBP‐Coatings are generally achieved from aqueous solutions at ambient temperature, and 1 g of LCI is reported to cover as a dense monolayer >600 m^2^ of a flat surface, which makes MBP‐coating a cost‐effective functionalization method.^[^ ^46^, ^47^, ^48^ ^]^ An LCI‐based biohybrid catalyst harboring a Grubbs‐Hoveyda (GH) type cofactor was reported to catalyze a ring‐opening metathesis polymerization (ROMP) of an oxanorbornene derivative to generate a hydrophilic polynorbornene film on the silica wafer and PP surface.^[^ ^49^ ^]^ Recently, a biohybrid catalyst harboring a Co(TACD) cofactor (TACD = 1,4,7,11‐tetraazacyclododecane) was shown to boost the hydroxylation of polystyrene (PS) microparticles using oxone as oxidant by directly immobilizing the cofactor on the PS particles through MBP LCI.^[^ ^50^ ^]^

In this study, we report a hydrophobic coating of Fe_3_O_4_ beads by a biohybrid catalyst employing the MBP LCI_F16C for the immobilization of the GH type cofactor onto Fe_3_O_4_ beads, catalyzing the ROMP reaction of a modified hydrophobic norbornene‐derivative (**Nor‐C18**). Hydrophobic film‐coated Fe_3_O_4_ beads were subsequently evaluated with respect to their ability to enrich various NPs, including polypropylene (PP), polyethylene (PE), polystyrene (PS), poly(methyl methacrylate) (PMMA), poly(ethylene terephthalate) (PET), and styrene‐butadiene rubber (SBR) from aqueous suspensions. The NP‐Fe_3_O_4_ aggregates were collected and removed from water by applying an external magnetic field for further determination of recovery rates and removal efficiency. GH cofactors with different spacer lengths (C3‐C10) were investigated to explore whether the linker length influences the performance of hydrophobic coatings to remove MPs/NPs. The captured SBR NPs were subjected to a further ethenolysis process for the cleavage of internal C═C bonds from the 1,4‐addition butadiene units in organic solvent in the presence of ethylene without any additional catalyst. The latter achieved, “Hooked for Decay” process, represents the first example of in situ degradation of untreated SBR nanoparticles via an ethenolysis process.

Grubbs‐Hoveyda cofactors **GH‐C3**, **GH‐C5**, and **GH‐C10** with varied spacer lengths of maleimide linker were synthesized and covalently bound through the cysteine residue at position 16 of the LCI_F16C variant by a Michael addition reaction in Tris‐HCl buffer (20 mM, pH 7.5, 150 mM NaCl) with 5% DMSO (v/v) (Figure 1a). Disulfide bond formation between LCI_F16C peptides was prevented via dithiothreitol (DTT) supplementation before GH‐cofactor conjugations. The structure of the biohybrid catalysts (**GH‐Cn**@LCI_F16C, n = 3, 5,10) was simulated by YASARA software (Figure 1b) based on the solved structure of LCI (PDB ID:2B9K^[^ ^51^ ^]^). Conjugation efficiencies of the GH cofactors for all three linker lengths in LCI_F16C were determined by inductively coupled plasma optical emission spectroscopy (ICP‐OES) and reached >90%, indicating that one GH‐cofactor was conjugated per LCI_F16C peptide (Table S1). The bioconjugation yield was further determined by cysteine titration using a maleimide‐bearing fluorescence indicator ThioGlo‐1, since the GH cofactors could be non‐specifically bound to the peptide surface through non‐covalent binding or other interactions (Figure 1c).^[^ ^52^, ^53^ ^]^ Upon addition of ThioGlo‐1 to the solution containing free LCI_F16C, the fluorescence intensity increased significantly compared to the control experiment without peptide. In contrast, the sample of **GH‐C3**@LCI_F16C, **GH‐C5**@LCI_F16C, and **GH‐C10**@LCI_F16C exhibited only slightly higher fluorescence intensity than the negative control, suggesting a coupling efficiency of approximately 90%, which confirms the results of ICP‐OES. Circular dichroism (CD) spectroscopy through the characteristic minimum at λ = 210 nm (Figure 1d) of LCI_F16C in Tris‐HCl buffer (50 mM, pH 8.0; at 25 °C) further confirmed the structural integrity of the biohybrid catalysts.

**Figure 1:**
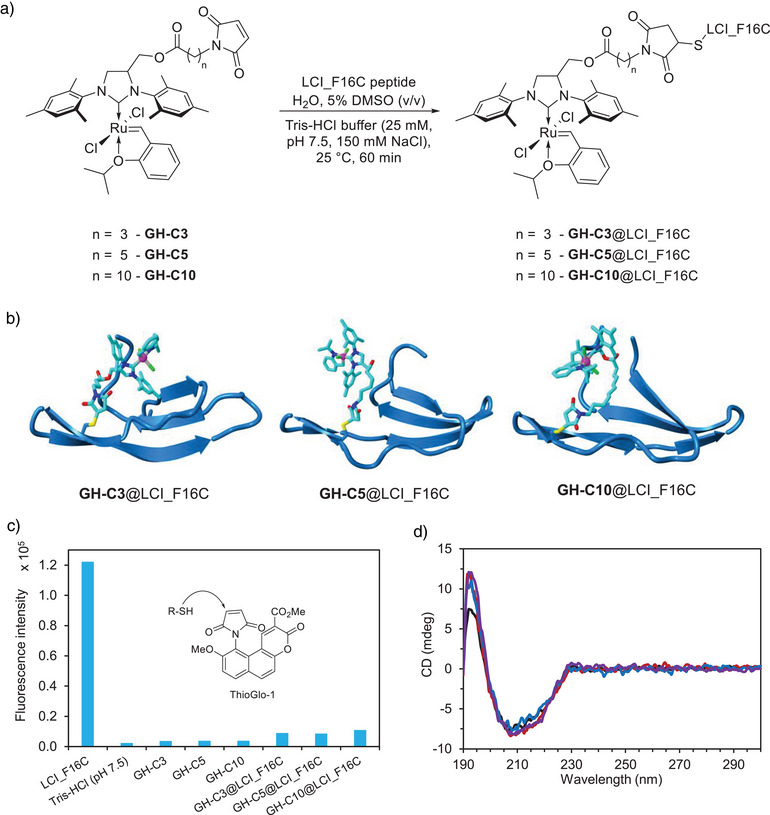
a) Conjugation of GH‐cofactors to LCI_F16C. b) Structure of **GH‐C3**@LCI_F16C, **GH‐C5**@LCI_F16C, and **GH‐C10**@LCI_F16C. c) Coupling of fluorescence indicator ThioGlo‐1 to the biohybrid catalysts to determine the content of non‐reacted ‐SH groups in LCI_F16C. Conditions: 10 µM LCI_F16C, 50 µM cofactor, Tris‐HCl buffer (20 mM, pH 7.5, 150 mM NaCl), 5% DMSO (v/v), 25 °C, 1 h with subsequent addition of 20 µM ThioGlo‐1. d) CD spectra of LCI_F16C (black), **GH‐C3**@LCI_F16C (red), **GH‐C5**@LCI_F16C (blue), and **GH‐C10**@LCI_F16C (violet) in Tris‐HCl buffer (20 mM, pH 7.5, 150 mM NaCl), indicating that GH‐cofactor conjugation did not affect the overall LCI_F16C structure.

A schematic illustration of the hydrophobic film‐coated SPION is presented in Figure 2a. The biohybrid catalyst **GH**‐C3@LCI_F16C is immobilized on the Fe_3_O_4_ surface via the binding of LCI_F16C peptide, while the conjugated GH‐cofactor initiates the ROMP reaction to form a hydrophobic polynorbornene outer film. Successful catalyst immobilization and polymer film formation were verified via fluorescent microscopy. A signal was observed through binding of the fluorescent streptavidin to the strep tag fused to the LCI peptide (Figure 2b, left), and the formation of the polymer film on the Fe_3_O_4_ surface was confirmed by employing a ‐SH group containing monomer (**Nor‐SH**) followed by conjugation with the ThioGlo‐1 dye (Figure 2b, right; Scheme S1). Thermogravimetric analysis (TGA) results demonstrated a clear stepwise increase in mass loss with each level of modification (6.5% for bare Fe_3_O_4_, 16.3% for **GH**‐C3@LCI_F16C@Fe_3_O_4_, and 47.1% for film‐coated Fe_3_O_4_), supporting the successful attachment of **GH**‐C3@LCI_F16C and subsequent polymer film formation. The presence of distinct peaks in the derivative thermogravimetric (DTG) curves further confirmed the introduction of organic components onto the SPION surfaces (Figure 2c). The spectrum obtained by X‐ray photoelectron spectroscopy (XPS) presented the chemical composition, with the appearance of N1s peak in **GH‐C3**@LCI_F16C@Fe**_3_** O**_4_** indicating LCI peptide attachment, and the enhanced C1s peak in film‐coated Fe_3_O_4_ reflecting polymer film formation (Figure 2d). Fourier‐transform infrared spectroscopy (FT‐IR) analysis provided insights into the surface functionalization of SPIONs; **GH‐C3**@LCI_F16C@Fe_3_O_4_ exhibited new peaks at 1248, 1539, and 1643 cm^−1^, corresponding to amide III, II, and I bands, respectively, indicating peptide attachment. Additionally, weak signals at 2909 and 2982 cm^−1^ were attributed to C─H stretching from aliphatic side chains of the peptide. In the case of film‐coated Fe_3_O_4_, the pronounced peaks at 2848 and 2915 cm^−1^ were assigned to the aliphatic C₁₈ hydrocarbon tails of the monomer, while the peak at 1697 cm^−1^ indicated C═O stretching from the monomer backbone, collectively confirming the successful formation of a hydrophobic polymer coating around the Fe_3_O_4_ core (Figure 2e). The polymer film (**poly‐Nor‐C18**) was characterized by ^1^H NMR, which revealed characteristic olefinic hydrogen peaks between 5.4 and 5.6 ppm (Figure S5). The vibrating sample magnetometer (VSM) showed that the magnetic moment and superparamagnetic behavior of the SPIONs are negligibly influenced by film decoration (Figure S6). Scanning electron microscope (SEM) images showed a smoother surface morphology following film decoration compared to bare iron oxide, with the decorated particles exhibiting a size of approximately 500 nm (Figure 2f and g).

**Figure 2:**
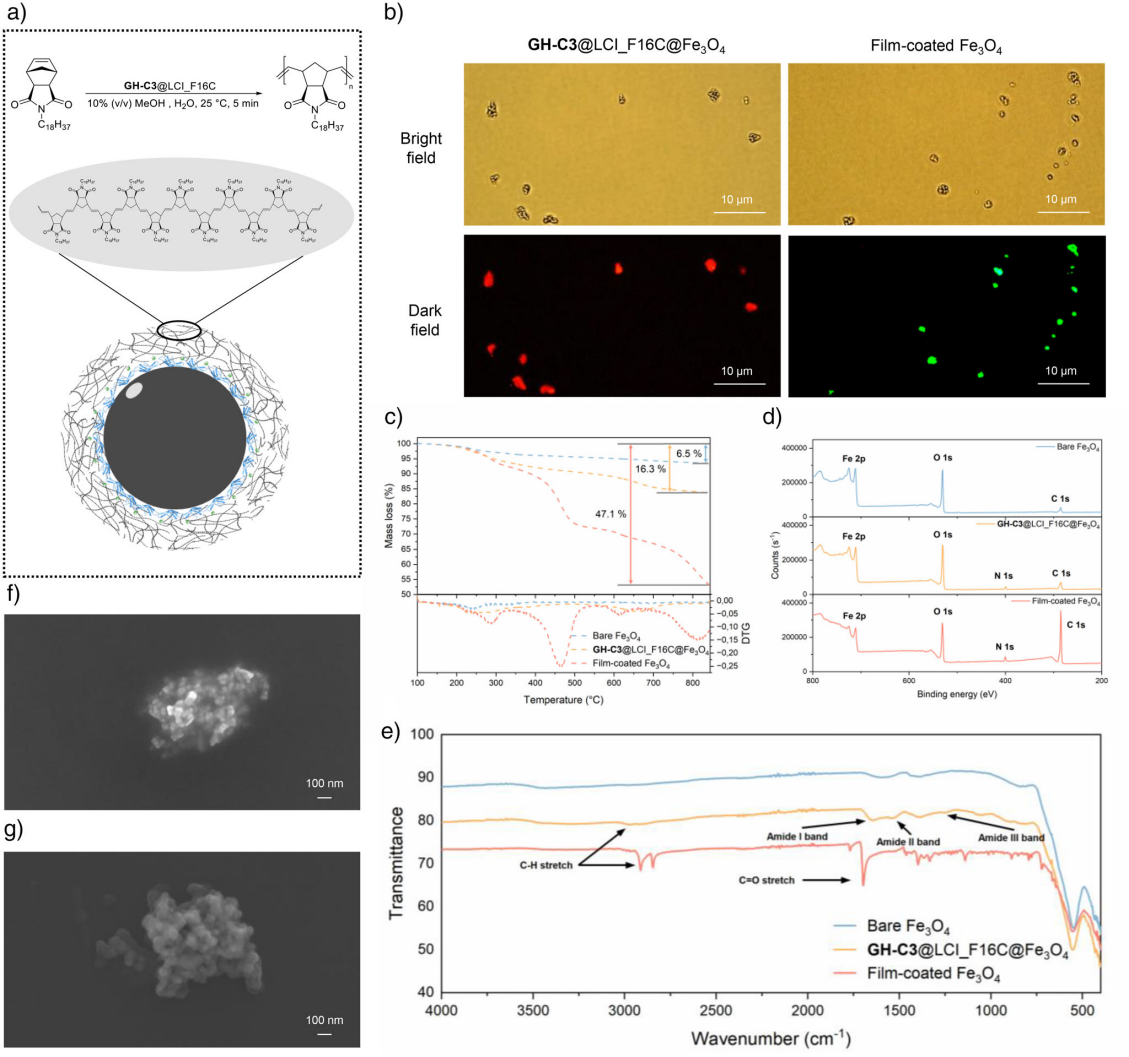
a) Scheme illustrating the decoration of a SPION with a hydrophobic polynorbornene film, achieved by immobilizing the GH cofactor, which initiates a ROMP reaction, through the material‐binding peptide LCI_F16C. b) Microscope images demonstrating the decoration of SPIONs with biohybrid catalyst **GH‐C3**@LCI_F16C (left) and with generated hydrophobic outer film (right). c) TGA measurements of bare Fe_3_O_4_ (blue), **GH‐C3**@LCI_F16C@Fe_3_O_4_ (orange), and film‐coated Fe_3_O_4_ (red). d) XPS spectra of bare Fe_3_O_4_ (top), **GH‐C3**@LCI_F16C@Fe_3_O_4_ (middle), and film‐coated Fe_3_O_4_ (bottom). e) FT‐IR spectrum of bare Fe_3_O_4_ (blue), **GH‐C3**@LCI_F16C@Fe_3_O_4_ (orange), and film‐coated Fe_3_O_4_ (red). SEM images of f) bare Fe_3_O_4_ nanoparticles, and g) film‐coated Fe_3_O_4_ nanoparticles. Protein models are visualized and colored by ChimeraX 1.4.^[^ ^54^ ^].^

To evaluate the performance of film‐coated Fe_3_O_4_ beads, enrichment experiments using PS‐COOH_500 nm_ NPs were carried out in 200 µL reaction volumes. As outlined in Figure 3a, a six‐step workflow was established to assess the enrichment efficiency of the developed platform, including an absorbance‐based quantification method for NPs. The optimal peptide concentration for Fe_3_O_4_ beads decoration was determined using fluorescence measurements, in which the LCI_F16C peptide (post‐DTT reduction) was incubated with bare Fe_3_O_4_ beads, and ThioGlo‐1 dye was used to quantify surface binding. An LCI_F16C concentration of 3 µM was found to be sufficient for saturated decoration of 0.075 g L^−1^ of iron oxide with 1 min shaking in the buffer system at ambient temperature (Figure S7). To fine‐tune monomer concentration, polymerization time, and linker length between the GH‐cofactor and LCI_F16C, enrichment experiments were conducted using 0.075 g L^−1^ Fe_3_O_4_ beads and 0.2 g L^−1^ PS‐COOH_500 nm_, in 100 mM NaCl with a 30s shaking period. As shown in Figure 3b, **GH‐C3**@LCI_F16C achieved optimal enrichment performance with a 500 µM monomer concentration and a five‐minute polymerization time, highlighting a faster functionalization process than previously reported SPION systems.^[^ ^26^, ^29^, ^34^ ^]^ Figure 3c shows the better enrichment performance of the biohybrid catalyst with GH‐cofactor containing shorter linker **GH‐C3**@LCI_F16C compared to C5 and C10. The increase of adsorption capacity with shorter linker length could be attributed to the higher catalytic activity of the cofactor GH(II)‐C3 by locating the ruthenium center closer to the surface of LCI_F16C.^[^ ^55^ ^]^ Overall, a 99% recovery of PS‐COOH_500 nm_ NPs could be achieved with film‐coated Fe_3_O_4_ beads in contrast to a 3% recovery of unmodified Fe_3_O_4_ beads (Figure S9).

**Figure 3:**
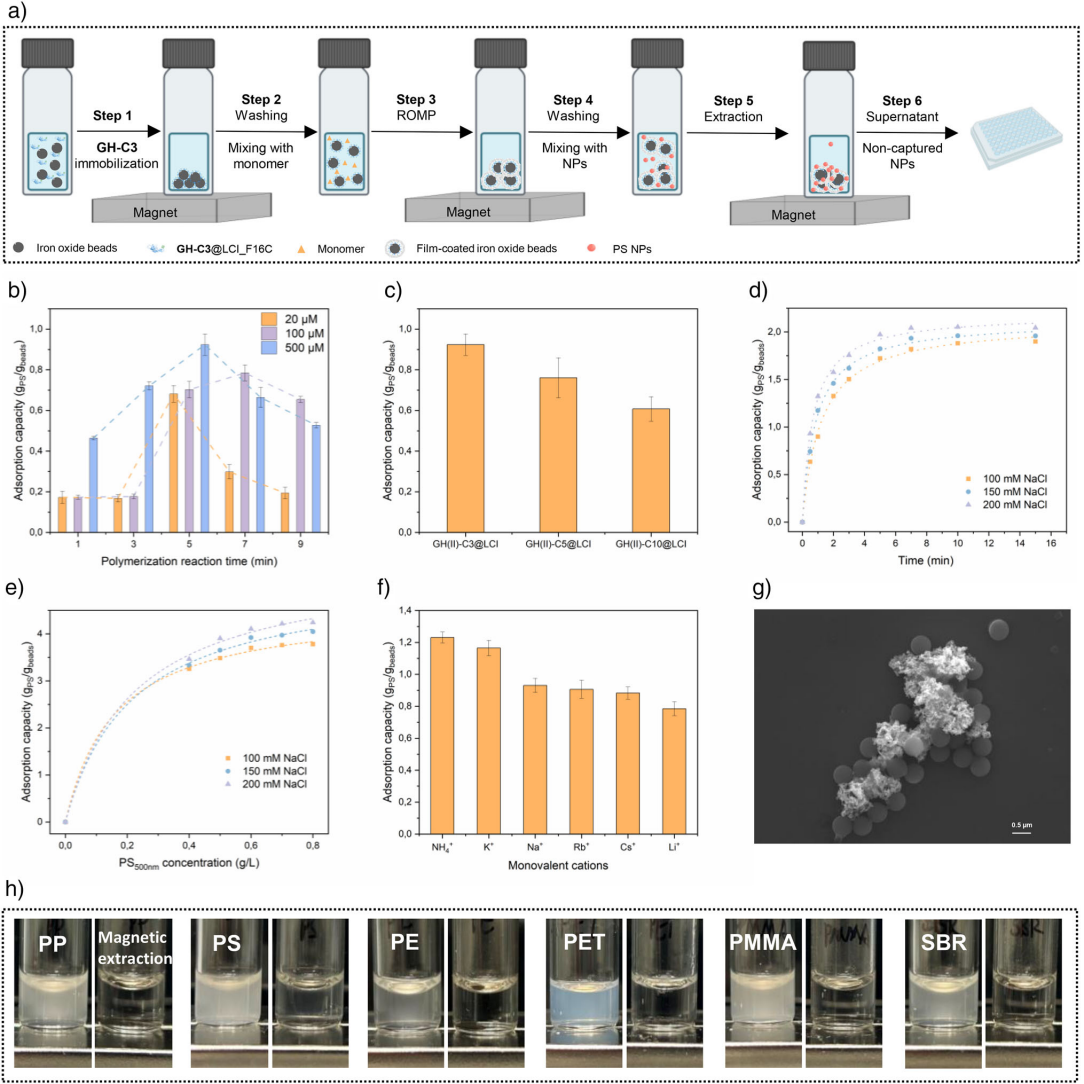
a) Scheme of the six‐step workflow developed for assessing the enrichment performance of the “Hooked for Decay” platform on a 200 µL scale. In Step 1, SPIONs were decorated with **GH‐C3**@LCI_F16C in 50 mM Tris, pH 8; in Step 2, the coated beads were washed with water, followed by mixing with monomer (**Nor‐C18**) in water; in Step 3, the further coated beads were washed again with water; in Step 4, the coated beads were mixed with NPs in the presence of NaCl; in Step 5, beads‐NPs complex was extracted by a magnet; and in Step 6, the supernatant containing non‐captured NPs was taken for absorbance measurement. The adsorption performance was determined in b) by increasing monomer concentration and polymerization time, and c) by increasing the linker length between the GH‐cofactor and LCI peptide. d) Adsorption kinetics to determine enrichment of PS‐COOH_500 nm_ NPs at varied salt concentrations (100, 150, and 200 mM) fitted with a pseudo‐second‐order model. e) Adsorption isotherm to determine enrichment of PS‐COOH_500 nm_ NPs at varied salt concentrations (100, 150, and 200 mM) fitted with the Langmuir model. f) Adsorption capacity of PS‐COOH_500 nm_ NPs by employing film‐coated beads in the presence of 100 mM monovalent cations (Li^+^, Na^+^, Cs^+^, Rb^+^, K^+^, and NH_4_^+^) during a 30‐s shaking period. g) SEM images of PS‐COOH_500 nm_ NPs with film‐coated Fe_3_O_4_ beads. h) Visualization of 0.2 g L^−1^ of dispersed PP, PS, PE, PET, PMMA, and SBR NPs before (left) and after magnetic extraction (right) by employing film‐coated Fe_3_O_4_ beads.

As salt concentration is known to influence hydrophobic interactions,^[^ ^56^ ^]^ the salting‐out effect of NaCl on the capture performance of hydrophobic film‐coated Fe_3_O_4_ beads towards PS‐COOH_500 nm_ NPs was systematically determined. High ionic strength of NaCl ions results in less frequent interactions of film‐coated Fe_3_O_4_ beads with water, resulting in hydrophobic interactions becoming the main driving force between film‐coated Fe_3_O_4_ beads and hydrophobic NPs.^[^ ^57^ ^]^ Adsorption kinetics and isotherms were used to analyze the NaCl effect. Kinetic studies revealed that the film‐coated Fe_3_O_4_ beads rapidly adsorbed PS‐COOH_500 nm_ NPs in the presence of 100 mM NaCl, reaching saturation within 10 min (Figure 3d). The adsorption followed a pseudo‐second‐order model that the interaction between adsorbate and adsorbent is the rate‐limiting step.^[^ ^58^ ^]^ Notably, the adsorption rate constant, *k*_2_, increased with NaCl concentration from 0.39 ± 0.02 g (g·min)^−1^ at 100 mM NaCl to 0.67 ± 0.05 g (g·min)^−1^ at 200 mM, nearly doubling the adsorption kinetics. The adsorption uptake capacity at equilibrium, q_e_, remained relatively stable, 2.10 ± 0.03 g/g, 2.12 ± 0.03 g/g versus 2.19 ± 0.03 g/g at 100 , 150 , and 200 mM NaCl, respectively (Table S3). Isotherm studies were conducted and fitted to Langmuir and Freundlich models to determine changes in adsorption capacity.^[^ ^59^ ^]^ The data closely followed the Langmuir model (*R* ^2^ > 0.99), indicating monolayer adsorption on a uniform surface (Figure 3e). The calculated maximum adsorption capacities, q_m_, increased with NaCl salt concentration, reaching 5.53 ± 0.29 g/g at 200 mM NaCl. The obtained capacities outperformed many previously reported systems, such as SPIONs‐PAC18 for PS (0.69 g/g) for PS NPs^[^ ^29^ ^]^ and iron oxide nanoflowers (1 g/g) for PE MPs.^[^ ^60^ ^]^ The addition of high salt concentration makes the “Hooked for Decay” system broadly applicable to water samples since hydrophobic interaction becomes the main driving force for binding of NPs on film‐coated Fe_3_O_4_ beads. Following the investigation of enrichment performances with 100 mM of various monovalent cations, a clear correlation to the Hofmeister series was observed with the following order NH_4_⁺ > K⁺ > Na⁺ > Rb⁺ > Cs⁺ > Li⁺ (Figure 3f).^[^ ^61^ ^]^ SEM imaging further confirmed agglomerates between film‐coated Fe_3_O_4_ beads and PS‐COOH_500 nm_ NPs (Figure 3g). Five additional NP types (PP, PE, PET, PMMA, and SBR; Figure S10) synthesized via emulsification–solvent evaporation^[^ ^62^ ^]^ and nanoprecipitation techniques^[^ ^63^ ^]^ spiked in ddH_2_O and PS NPs spiked in four kinds of environmental water samples (wastewater, lake, river, and sea) were investigated to validate the versatile applicability of film‐coated Fe_3_O_4_. All NPs suspensions turned clear after exposure to film‐coated Fe_3_O_4_ within 10 min (Figures 3h and S11), indicating efficient magnetic removal and versatile applicability of the “Hooked for Decay” platform.

SBR NPs were further degraded via ethenolysis catalyzed by the biohybrid catalyst (**GH‐C3**@LCI_F16C) after the enrichment due to its internal C═C bonds (Figure 4a). In this process, microaggregates obtained by enriching SBR nanoparticles with film‐coated Fe_3_O_4_ beads were directly added to a mixture of organic solvents (*p*‐xylene/THF, 1:1) to dissolve the enriched SBR NPs (for details see Supporting Information). Upon dissolution of SBR NPs and the polynorbornene film, the biohybrid catalyst (**GH‐C3**@LCI_F16C) is exposed, allowing it to bind with the SBR polymer as a substrate. The initial pressure of ethylene was set to 25 bars to ensure sufficient concentration of ethylene during the reaction. The ethylene pressure was monitored over 12 h, in which a slight decrease in pressure was observed, indicating ethylene consumption (Figure 4b). After the ethenolysis process, the biohybrid catalyst immobilized on SPION beads was efficiently removed by simple exposure to an external magnetic field. Degradation products were subsequently quantified by ^1^H NMR spectroscopy (Figure 4c). Compared to the untreated SBR polymer, new peaks corresponding to the protons of terminal C═C bonds were observed in the range of 4.9 – 4.5 ppm, which indicates the formation of terminal C═C bonds. The degradation efficiency of SBR NPs was calculated from the ^1^H NMR spectra ranging from 4.1% to 6.5% with 0.1 and 0.2 mol% catalyst loading in correspondence to the 1,4‐butadiene units, respectively. The biohybrid catalyst retains its activity for the ethenolysis reaction, probably because of the protection of the GH‐cofactor (**GH‐C3**) by the formed hydrophobic polymer film, minimizing contact with salt ions.^[^ ^64^, ^65^, ^66^, ^67^ ^]^ The latter results demonstrate the biohybrid catalyst's (**GH‐C3**@LCI_F16C) stability, double catalytic functionality in enrichment (Hook) and degradation (Decay).

**Figure 4:**
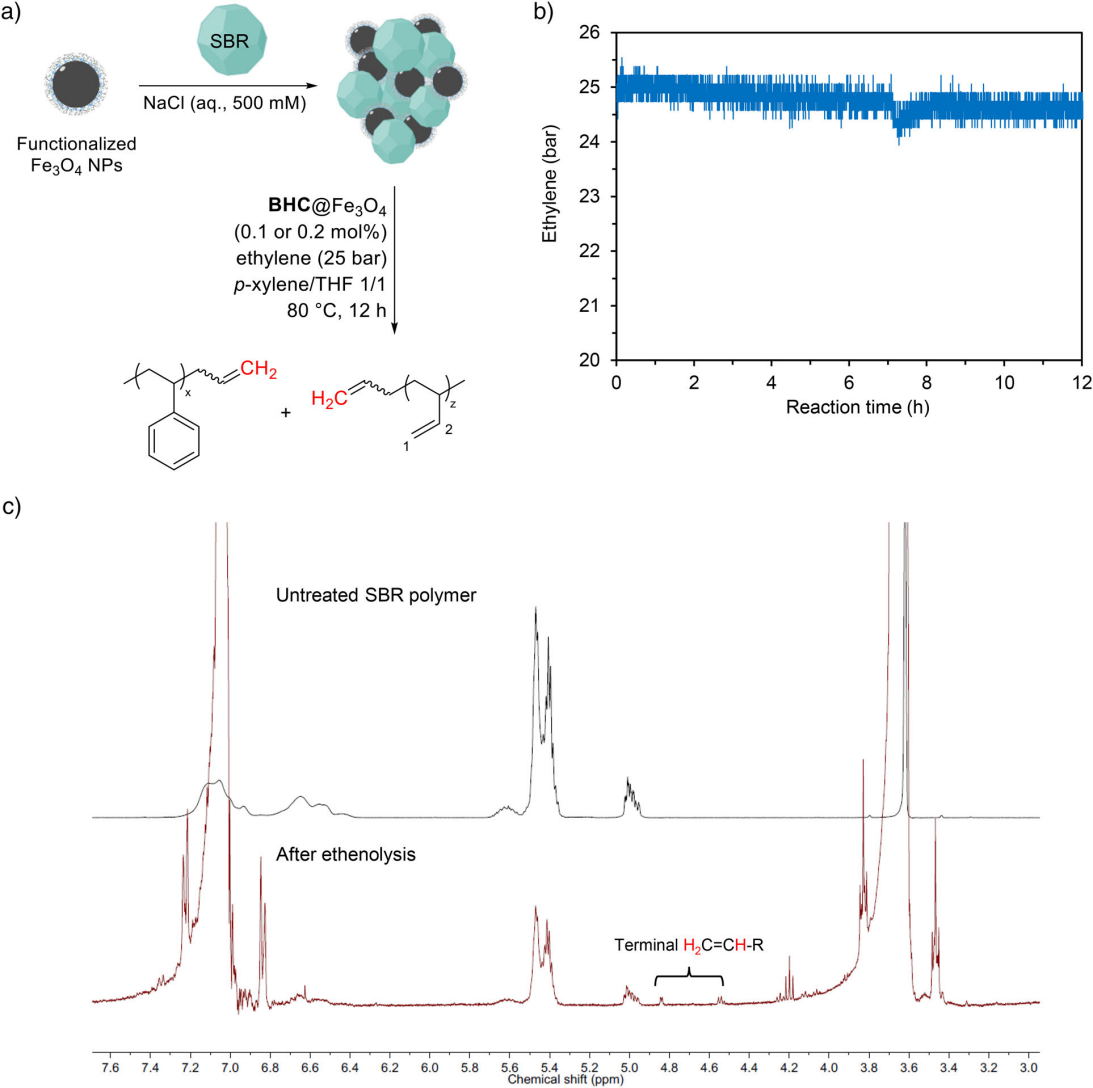
a) Enrichment of SBR nanoparticles using film‐coated Fe_3_O_4_ beads with subsequent ethenolysis reaction catalyzed by **GH‐C3**@LCI_F16C to break down the internal C═C bonds. b) Pressure monitoring during the ethenolysis process. c) ^1^H NMR spectra of SBR polymer before (top) and after (bottom) ethenolysis, confirming the formation of terminal C═C bonds.

In conclusion, a bifunctional “Hooked for Decay” biohybrid catalyst (**GH‐C3**@LCI_F16C) composed of a Grubbs‐Hoveyda cofactor and a MBP variant of LCI was found to be suitable for the efficient capture and removal of a wide range of hydrophobic NPs, including PP, PE, PS, PET, PMMA, and SBR. The hydrophobic polynorbornene‐coated Fe_3_O_4_ enabled rapid adsorption of PS NPs within 10 min of shaking and exhibited a high adsorption capacity of 5.53 ± 0.29 g/g for PS‐COOH_500 nm_, surpassing other known Fe_3_O_4_‐based materials. The 99% recovery of NPs was achieved in the presence of 100 mM NaCl, in which hydrophobic interaction becomes the main driving force for film‐coated Fe_3_O_4_‐NPs binding, making this platform broadly applicable for NPs removal from all kinds of aqueous solutions. The dual‐functionality of the Grubbs‐Hoveyda cofactor for the polymer film formation and ethenolysis (6.5% degradation) of SBR NPs demonstrates the potential for the removal and breakdown of synthetic rubber MP/NPs. The film‐coated Fe_3_O_4_ beads introduce a novel concept for the removal of hydrophobic NP‐pollutants from aqueous solutions. We believe that the use of a broad array of available monomers and MBPs constitutes a valuable technology platform to tackle challenges in plastic pollution in the environment and for human health.

## Supporting Information

The authors have cited additional references within the Supporting Information.^[^ ^68^, ^69^, ^70^, ^71^, ^72^, ^73^, ^74^, ^75^, ^76^, ^77^ ^]^

## Conflict of Interests

The authors declare no conflict of interest.

## Supporting information



Supporting Information

## Data Availability

The data that support the findings of this study are available from the corresponding author upon reasonable request.
