# Supplementary material for: Hooked for Decay with Hydrophobic‐Coated Magnetic Beads to Grapple and Disintegrate Nanoplastics
Source: Angew Chem Int Ed Engl. 2025 Aug 19;64(40):e202510356. doi: 10.1002/anie.202510356 (PMC12462746; doi:10.1002/anie.202510356)
Supplement: Supplementary file 1 — Supporting Information [file ANIE-64-e202510356-s001.docx]

Supporting Information

**Hooked for Decay with Hydrophobic-coated Magnetic Beads to Grapple and Disintegrate Nanoplastics**

Dong Wang,^+[a]^ Maochao Mao,^+[a]^ Maximilian Lorberg,^[b]^ Julian Luka,^[a]^ Marian Bienstein,^[a]^ Jun Okuda,^[c]^ and Ulrich Schwaneberg*^[a]^

[a] Dr. D. Wang, M. Mao, J. Luka, Dr. M. Bienstein, Prof. Dr. U. Schwaneberg
Institute of Biotechnology
RWTH Aachen University
Worringerweg 3, 52074 Aachen, Germany
E-mail: u.schwaneberg@biotec.rwth-aachen.de

[b] M. Lorberg
Institute of Technical and Macromolecular Chemistry
RWTH Aachen University
Worringerweg 2, 52074 Aachen, Germany

[c] Prof. Dr. J. Okuda
Institute of Inorganic Chemistry
RWTH Aachen University
Landoltweg 1, 52074 Aachen, Germany

+ These authors contributed equally

Table of Contents

[I. Experimental Section 2](#_Toc203560940)

[I.1 General remarks 2](#_Toc203560941)

[I.2 Protein modeling 2](#_Toc203560942)

[I.3 Protein purification 3](#_Toc203560943)

[I.4 Bioconjugation 3](#_Toc203560944)

[I.5 NMR spectroscopy 3](#_Toc203560945)

[I.6 CD spectroscopy 4](#_Toc203560946)

[I.7 FT-IR spectroscopy 4](#_Toc203560947)

[I.8 Inductively coupled plasma optical emission spectroscopy (ICP-OES) 4](#_Toc203560948)

[I.9 Preparation of film-coated Fe_3_O_4_ nanoparticles 4](#_Toc203560949)

[I.10 Preparation of fluorescence-labeled Fe_3_O_4_ nanoparticles 5](#_Toc203560950)

[I.11 Fluorescence microscope 5](#_Toc203560951)

[I.12 TGA measurement 5](#_Toc203560952)

[I.13 XPS spectrum 5](#_Toc203560953)

[I.14 FTIR spectrum 6](#_Toc203560954)

[I.15 Magnetization property measurement 6](#_Toc203560955)

[I.16 SEM imaging 6](#_Toc203560956)

[I.17 Enrichment of NPs with coated Fe_3_O_4_ nanoparticles 6](#_Toc203560957)

[I.18 Kinetic and isotherm models 7](#_Toc203560958)

[I.19 Preparation of PP, PE, and SBR nanoplastics 7](#_Toc203560959)

[I.20 Preparation of PET nanoplastics 7](#_Toc203560960)

[I.21 Degradation of SBR polymer via ethenolysis 8](#_Toc203560961)

[II. Synthesis of the cofactors 9](#_Toc203560962)

[III. Experimental Spectra 13](#_Toc203560963)

[IV. Supplementary Figures and Tables 18](#_Toc203560964)

[V. DNA Sequence of LCI_F16C 25](#_Toc203560965)

[VI. References 26](#_Toc203560966)

# Experimental Section

## General remarks

All chemicals were obtained from commercial suppliers (*Sigma-Aldrich/Merck, TCI, BLDpharm,* and others), unless otherwise noted. The experiments were performed under an inert atmosphere of nitrogen or argon using standard Schlenk or glovebox techniques. The deionized water was bubbled with argon (60 min) prior to use. Other solvents were degassed by the “freeze-pump-thaw” technique. Dichloromethane, diethyl ether, pentane, THF and toluene were obtained dry and degassed from an SPS 800 from MBraun. Acetonitrile, acetonitrile-*d*3 dichloromethane-*d*2 and chloroform-*d*1 were dried over calcium hydride, distilled, degassed and stored in a glove box. **GH-OH** and **Nor-SH** were synthesized according to a previously reported procedure.^[1,2]^ GH type cofactors (**GH-C3**, **GH-C5**, and **GH-C10**) and the maleimide precursors (**Mal-C3**, **Mal-C5**, and **Mal-C10**) were synthesized according to a modified procedure.^[1,3]^

## Protein modeling

As a structural basis for the modeling of the biohybrid catalysts, an NMR solution structure of wild-type LCI (PDB: 2B9K) was used. The F16C mutation, introducing the anchoring cysteine residue, was performed *in silico*. The mutated LCI_F16C was subjected to energy minimization using YASARA^[4]^ Structure Vers. 20.12.24, employing force field AMBER14.^[5]^ According to the previously published procedure,^[6,7]^ the modeling was carried out using YASARA Structure Vers. 20.12.24 employing force field AMBER14 for protein residues and GAFF^[8]^ using AM1/BCC^[9]^ partial charges for the catalyst covalently bound to Cys16. To maintain the correct coordination geometry, the distances and angles from the metal to all coordinating atom were constrained according to the previously reported crystal structure.^[10]^ Additional atoms were added manually in the linking unit. The charge of the metal was set to +2, and the total charge of the catalyst was set to 0. The linker was placed manually adjacent to Cys16 and a bond from Cys S atom to the C1 atom of the maleimide group was defined. The constructed biohybrid catalysts were solvated in a box of TIP3P water molecules using periodic boundaries at pH 7 and a density of 0.997 g/mL. Three starting structures were analyzed and favorable models were identified for covalent attachment to the reactive maleimide atoms by steepest descent minimization and simulated annealing. The pre-minimized structures were relaxed using molecular dynamics calculations at 298 K for 5000 ps and snapshots were taken every 25 ps to analyze the binding modes.

## Protein purification

LCI_F16C with N-terminal strep-tag was expressed in Corynebacterium glutamicum ATCC 13032. The cultivation of pre-cultures was performed in 5 mL Brain heart infusion-supplemented (BHIS) medium (37 g/L brain heart infusion powder, 91 g/L sorbitol, 50 μg/mL kanamycin) using a 15 mL Erlenmeyer flask (30 °C, 200 rpm, 70 % humidity, 24 h; Multitron II, Infors GmbH, Einsbach, Germany). Main cultures (200 mL BHIS medium supplemented with 25 μg/mL kanamycin in a 1 L Erlenmeyer flask) were inoculated using pre-cultures as inoculum to an OD600 of 0.05. Main cultures were incubated at 30 °C, 250 rpm, and when the OD600 reached 0.6–1.0, they were induced with 0.1 mM IPTG. After cultivation (18 °C, 200 rpm, 70% humidity, 48 h; Multitron II, Infors GmbH), the culture supernatant was separated from cells by centrifugation (Sorvall, ThermoFischer Scientific, Darmstadt, Germany; 4 °C, 5000 rpm, 30 min). The culture supernatant (200 mL) was filtered (0.45 μm) and applied to a 5 mL Strep-Tactin® Sepharose® (IBA Life Sciences) column to purify the target protein (washing buffer: 50 mM Tris-HCl, 300 mM NaCl, pH 8; elution buffer: 50 mM Tris-HCl, 300 mM NaCl, 2.5 mM desthiobiotin, pH 8). Finally, the eluted protein was applied to a PD 10 desalting column (Cytiva) for buffer exchange and stored in Tris-HCl buffer (50 mM, pH 8.0).

## Bioconjugation

The conjugation of Grubbs-Hoveyda type cofactors (**GH-C3**, **GH-C5**, and **GH-C10**) to LCI_F16C was achieved by covalent binding of maleimide linker to the reduced thiol group of cysteine within LCI_F16C. The disulfide bond was reduced by incubating LCI_F16C (200 µM) with DTT (15 mM) for 60 min at room temperature. Subsequently, DTT was removed by buffer exchange, and the GH-cofactors were further conjugated to LCI_F16C by incubating the peptide with 5.0 equiv. of cofactor (final concentrations: 20 µM protein, 100 µM cofactor) in Tris-HCl buffer (20 mM, pH 7.5, 150 mM NaCl) with 5% DMSO (v/v) for 60 min at room temperature. After the conjugation, the buffer was exchanged to the binding buffer (Tris-HCl, 50 mM, pH 8.0) to store the biohybrid catalysts (**GH-C3**@LCI_F16C, **GH-C5**@LCI_F16C, and **GH-C10**@LCI_F16C).

## NMR spectroscopy

NMR measurements were performed on a Bruker Avance II or Avance III spectrometer operating at 400 MHz for ^1^H nuclei and 101 MHz for ^13^C nuclei. The chemical shifts (in ppm, parts per million) of the ^1^H and ^13^C NMR spectra were referenced to the residual proton signals of the deuterated solvents and reported relative to tetramethylsilane. Standard abbreviations indicating multiplicities were used as follows: s (singlet), d (doublet), t (triplet), q (quartet), quint (quintet), and m (multiplet).

## CD spectroscopy

Circular dichroism (CD) spectra were recorded on a *JASCO J-1100* spectrometer equipped with a single-position Peltier cell holder. If not stated otherwise, the temperature was set to 19 °C. The path length of the cuvette was 0.2 mm. The protein concentration was 20 µM.

## FT-IR spectroscopy

FT-IR spectra were performed in powder form using *Nicolet iS20*, Thermo Scientific.

## Inductively coupled plasma optical emission spectroscopy (ICP-OES)

ICP-OES measurements were conducted on a Spectro Analytical Instruments Spectroblue ICP-OES spectrometer. A serial dilution was prepared by using a commercially available ruthenium standard (1.0 g/L, Sigma-Aldrich). The calibration was done with samples containing 0 ppm, 2 ppm, and 5 ppm Ru. The results of the measurements are shown in the following **Table S1**.

Table S1. ICP-OES results for conjugation of GH-cofactors to LCI_F16C. The SH group in cysteine 16 was blocked by supplementing ThioGlo-1 to LCI_F16C, which was confirmed by fluorescence measurement. After the addition of cofactor as well as the purification, ICP results of the blocked LCI sample did not show significantly more Ru than that of the negative control with the buffer solution.

| Entry | Catalyst | Expected Ru content (ppm) | Measured Ru content (ppm) | Conjugation efficiency |
| --- | --- | --- | --- | --- |
| 1 | no catalyst | 0.00 | 0.05 | --- |
| 2 | **GH-C3**@LCI_F16C | 3.61 | 3.27 ± 0.05 | 91% |
| 3 | **GH-C5**@LCI_F16C | 3.48 | 3.12 ± 0.05 | 90% |
| 4 | **GH-C10**@LCI_F16C | 3.96 | 3.51 ± 0.05 | 89% |

## Preparation of film-coated Fe_3_O_4_ nanoparticles

The Immobilization of biohybrid catalyst was carried out by mixing Fe_3_O_4_ nanoparticles (75 µg/mL) with an excess amount of catalyst (5 µM) in Tris-HCl buffer (50 mM, pH 8.0). Total volume = 200 µL. The mixture was shaken for 1 min, and the peptide-functionalized Fe_3_O_4_ nanoparticles were washed twice with water to remove the non-bound biohybrid catalyst. After resuspending the particles in water, the modified monomer (20 – 500 µM, 10 mM stock in methanol) was added, and the mixture was shaken for 1 to 9 min to form the hydrophobic film. The film-coated Fe_3_O_4_ nanoparticles were washed twice with water to remove the unreacted monomer and resuspended in water for later use.

## Preparation of fluorescence-labeled Fe_3_O_4_ nanoparticles

Scheme S1. ROMP of Nor-SH with following labeling using ThioGlo-1.

The formation of polymer film was carried out with **Nor-SH** as monomer according to the procedure described above. After the polymerization, ThioGlo-1 was added to label the thiol-group for further fluorescence characterization.

## Fluorescence microscope

The fluorescence microscope (BX51, Olympus) was used to check the binding of peptides and hydrophobic film formation, with streptavidin-conjugated Alexa fluorophore that recognizes strep-tag in LCI_F16C peptide and ThioGlo-1 dye that conjugates with the introduced free thiol groups in the monomer.

## TGA measurement

The thermogravimetric analysis (TGA; STA6000, PerkinElmer) was used to examine the mass loss of samples during temperature increase from 100 °C to 800 °C.

## XPS spectrum

The X-ray photoelectron spectroscopy (XPS; AXIS supra+, Kratos Analytical) was used to determine the chemical composition of functionalized Fe_3_O_4_ nanoparticles.

## FTIR spectrum

The Fourier-transform infrared spectroscopy (FTIR; Nicolet iS20, Thermo Scientific) was used to study the surface chemistry of functionalized Fe_3_O_4_ nanoparticles.

## Magnetization property measurement

The SQUID magnetometer (MPMS-XL, Quantum Design) was used to study the magnetic properties of the beads before and after functionalization.

## SEM imaging

A scanning electron microscope (SEM; SU9000, Hitachi) was used to study the morphology of the beads before and after functionalization.

## Enrichment of NPs with coated Fe_3_O_4_ nanoparticles

Enrichment on a 200 µL scale was done in the glass vial and characterized by UV absorbance measurement (CLARIOstar, BMG Labtech). Specifically, the prepared film-coated beads were mixed with 0.2 g/L of PS NPs and 100-200 mM NaCl in a water solution at a total volume of 200 µL. Followed by shaking at 1200 rpm for 20 min and magnetic extraction, 100 µL of supernatant was transferred into a 96-well plate for absorbance characterization. The concentration was obtained according to the UV-vis standard curves, and the NPs recovery rate (*R*_1_) was calculated using equation (1),

$R_{1}=\left( 1-\frac{C_{t}}{C_{0}} \right)*100\%$ (1)

where *C*_t_ represents the NPs concentration in the supernatant after magnetic extraction, and *C*_0_ is the concentration of the original NPs water dispersion. The adsorption uptake *q* (g/g) was calculated using equation (2),

$q=\frac{C_{0}-C_{t}}{C_{m}}$ (2)

where *C*_m_ is the concentration of film-coated beads. Each experiment was done in triplicate.

## Kinetic and isotherm models

The adsorption kinetic process was described by two adsorption models in this study, including the Pseudo-first-order (3) and Pseudo-second-order (4).^[11]^

$q_{t}=q_{e}(1-e^{-k_{1}t})$ (3)

$q_{t}=\frac{k_{2}q_{e}^{2}t}{1+k_{2}q_{e}t}$ (4)

The *q*_t_ and *q*_e_ are the amounts of NPs adsorbed per unit mass of film-coated beads at time, *t,* and at equilibrium, respectively; *k*_1_ and *k*_2_ are adsorption rate constants for the Pseudo-first-order and the Pseudo-second-order, respectively.

The adsorption isotherm was described by two adsorption models in this study, including the Freundlich model (5) and Langmuir model (6).^[12]^

$q_{e}=K_{F}C_{e}^{\frac{1}{n}}$ (5)

$q_{e}=\frac{q_{m}K_{L}C_{e}}{1+K_{L}C_{e}}$ (6)

The *K*_F_ (L/g) and *K*_L_ (L/g) are the isotherm constants for the Freundlich and Langmuir models, respectively; *C*_e_ (g/L) is the concentration of adsorbate, PS NPs; *q*_m_ and *q*_e_ are the adsorption capacity at maximum and equilibrium, respectively.

## Preparation of PP, PE, and SBR nanoplastics

PP, PE, and SBR nanoparticles were synthesized as described by Faeze *et al.*^[13]^ with modifications. Briefly, 10 mg of PP granules, LD-PE powder, or SBR granules were dissolved in 5 mL of xylene at 110 °C in a glass beaker on a heater for 10 minutes until the solids were completely dissolved. With vigorous stirring, 20 mL of icy deionized water was rapidly added to emulsify the mixture, followed by immediate water bath sonication for 30 minutes. Once the PP/PE/SBR particles were suspended in water, Whatman Grade 1 qualitative filter paper was used to filter out the larger particles. The filtration process was repeated one more time after mild magnetic stirring for 2 hours at room temperature to obtain the nanoparticle dispersion in water.

## Preparation of PET nanoplastics

PET nanoparticles were prepared according to a reported protocol.^[14]^ Briefly, 10 mg of PET powder was dissolved in 2 mL 1,1,1,3,3,3-hexafluoro-2-propanol at room temperature for 1 h. The PET solution was dropped into 20 mL of deionized water with vigorous magnetic stirring. The organic solvent was removed from the particle suspension by continuous mild stirring for 2 hours at room temperature, followed by paper filtration to separate the precipitated particles.

## Degradation of SBR polymer via ethenolysis

For the ethenolysis process, an up-scaled enrichment of SBR particles was performed. The prepared SBR particles (**x** mg) were mixed with coated Fe_3_O_4_ nanoparticles (**y** mg) in a sodium chloride solution (10 mL, 500 mM). After shaking the mixture for 10 min, the microaggregates were collected by applying an external magnetic field. The collected microaggregates were transformed into THF/*p*-xylene 1/1 (4 mL) and sealed in a finger-autoclave. Ethylene (25 bar) was charged under stirring till the pressure was stable. The mixture was stirred at 80 °C for 12 h. After the removal of Fe_3_O_4_ particles, the solution was characterized by ^1^H-NMR spectroscopy.

Table S2. Summary of reaction conditions.

| Entry | **x**^a^ [mg] | **y** [mg] | Catalyst loading^c^ [mol%] | Conversion^d^ [%] |
| --- | --- | --- | --- | --- |
| 1 | 15.1 | 4 | 0.1 | 4.1 |
| 2 | 15.1 | 8 | 0.2 | 6.5 |
| ^a^Determined by measuring the mass after lyophilization. ^c^Calculated based on the 1,4-addition butadiene units. ^d^Calculated by ^1^H-NMR | | | | |

# Synthesis of the cofactors

Scheme S2. Synthetic route for the synthesis of GH type cofactors.

**General procedure for the synthesis of maleimide precursors (Mal-Cn-COCl):^[3]^**

The corresponding carboxylic acid (5 mmol) was dissolved in THF (25 mL). Thionyl chloride (2.8 mL, 37.5 mmol) was added dropwise, and the solution was stirred at room temperature for 16 h. The solution was concentrated under reduced pressure and the product was crystallized from THF at -40°C to give the corresponding product.

*4-(2,5-dioxo-2,5-dihydro-1H-pyrrol-1-yl)butanoyl chloride* (**Mal-C3-COCl**)

Colorless solid; yield: 67%; ^1^H-NMR (400 MHz, CD_2_Cl_2_, 298 K): δ 6.71 (s, 2H), 3.57 (t, 2H), 2.94 (t, 2H), 1.96 (qu, 2H).

*6-(2,5-dioxo-2,5-dihydro-1H-pyrrol-1-yl)hexanoyl chloride* (**Mal-C5-COCl**)

Pale yellow oil; yield: 55%; ^1^H-NMR (400 MHz, CD_2_Cl_2_, 298 K): δ 6.68 (s, 2H), 3.49 (t, 2H), 2.91 (t, 2H), 1.76 – 1.54 (m, 4H), 1.38 – 1.29 (m, 2H).

*11-(2,5-dioxo-2,5-dihydro-1H-pyrrol-1-yl)undecanoyl chloride* (**Mal-C10-COCl**)

Pale yellow oil; yield: 56%; ^1^H-NMR (400 MHz, CD_2_Cl_2_, 298 K): δ 6.66 (s, 2H), 3.47 (t, 2H), 2.89 (t, 2H), 1.74 – 1.49 (m, 4H), 1.41 – 1.17 (m, 12H).

**General procedure for the synthesis of GH type cofactors:^[1]^**

In an argon filled glovebox, **GH-OH** (33 mg, 0.05 mmol) was dissolved in dichloromethane (1 mL). Diisopropylethylamine (13 µL, 0.076 mmol) and **Mal-Cn-COCl** (0.075 mmol) were added to the solution. The solution was stirred at room temperature for 20 min. The solvent was evaporated, and the dark green oil was purified by column chromatography (silica gel, gradient of ethyl acetate/*n*-hexane from 1/10 to 1/4) to give the product as a green solid.

**GH-C3**

Green solid; yield: 60%; ^1^H NMR (400 MHz, CD_2_Cl_2_, 298 K): δ 16.42 (s, 1H), 7.59 – 7.53 (m, 1H), 7.17 – 6.99 (m, 4H), 6.98 – 6.82 (m, 3H), 6.67 (s, 2H), 4.96 – 4.82 (m, 1H), 4.76 – 4.60 (m, 1H), 4.32 (t, J = 10.6 Hz, 1H), 4.23 – 4.17 (m, 2H), 4.03 – 3.97 (m, 1H), 3.50 (t, J = 6.9 Hz, 2H), 2.56 – 2.24 (m, 20H), 1.83 (quint, J = 7.1 Hz, 2H), 1.27 – 1.20 (m, 6H).

ESI-MS(+): *m/z* calculated for C_40_H_47_ClN_3_O_5_Ru^+^ [M-Cl]^+^ 786.2243; found 786.2245.

**GH-C5**

Green solid; yield: 53%; ^1^H NMR (400 MHz, CD_2_Cl_2_, 298 K): δ 16.42 (s, 1H), 7.59 – 7.51 (m, 1H), 7.18 – 6.99 (m, 4H), 6.96 – 6.82 (m, 3H), 6.65 (s, 2H), 4.96 – 4.82 (m, 1H), 4.72 – 4.63 (m, 1H), 4.30 (t, J = 11.0 Hz, 1H), 4.23 – 4.13 (m, 2H), 4.02 – 3.95 (m, 1H), 3.47 (t, 7.1 Hz, 2H), 2.57 – 2.23 (m, 20H), 1.65 – 1.50 (m, 6H), 1.34 – 1.16 (m, 6H).

ESI-MS(+): *m/z* calculated for C_42_H_51_ClN_3_O_5_Ru^+^ [M-Cl]^+^ 814.2556; found 814.2560.

**GH-C10**

Green solid; yield: 46%; ^1^H NMR (400 MHz, CD_2_Cl_2_, 298 K): δ 16.41 (s, 1H), 7.60 – 7.51 (m, 1H), 7.18 – 6.99 (m, 4H), 6.96 – 6.82 (m, 3H), 6.66 (s, 2H), 4.98 – 4.80 (m, 1H), 4.76 – 4.60 (m, 1H), 4.23 – 4.11 (m, 2H), 4.07 – 3.92 (m, 2H), 3.46 (t, J = 7.2 Hz, 2H), 2.53 – 2.20 (m, 20H), 1.64 – 1.46 (m, 6H), 1.30 – 1.22 (m, 16H).

ESI-MS(+): *m/z* calculated for C_47_H_61_ClN_3_O_5_Ru^+^ [M-Cl]^+^ 884.3388; found 884.3389.

**Synthesis of Nor-C18:**

3a,4,7,7a-Tetrahydro-1H-4,7-methanoisoindole-1,3(2H)-dione (1.63 g, 10 mmol, *endo-* and *exo-* mixture) was suspended in MeCN (50 mL). 1-bromooctadecane (5.00 g, 15 mmol) and K_2_CO_3_ (2.07 g, 15 mmol) were added to the suspension. The mixture was stirred at 65 °C for 72 h. After cooling, the suspension was mixed with water (50 mL), and the product was extracted with DCM (50 mL, 3x). The organic layers were combined, dried over MgSO_4_, and concentrated under reduced pressure. The resulting oil was purified by column chromatography (silica gel, ethyl acetate/*n*-hexane 5/1) to give the *exo*- product as a colorless solid (1.61 g, 3.87 mmol, 77%).

^1^H NMR (400 MHz, CD_2_Cl_2_, 298 K): δ 6.44 – 6.19 (m, 2H), 3.48 – 3.43 (m, 2H), 3.30 – 3.20 (m, 2H), 2.74 – 2.61 (m, 2H), 1.59 – 1.49 (m, 3H), 1.36 – 1.25 (m, 31H), 0.92 (t, J = 6.8 Hz, 3H).

ESI-MS(+): *m/z* calculated for C_27_H_46_NO_2_^+^ [M-H]^+^: 416.3524; found 416.3522.

# Experimental Spectra


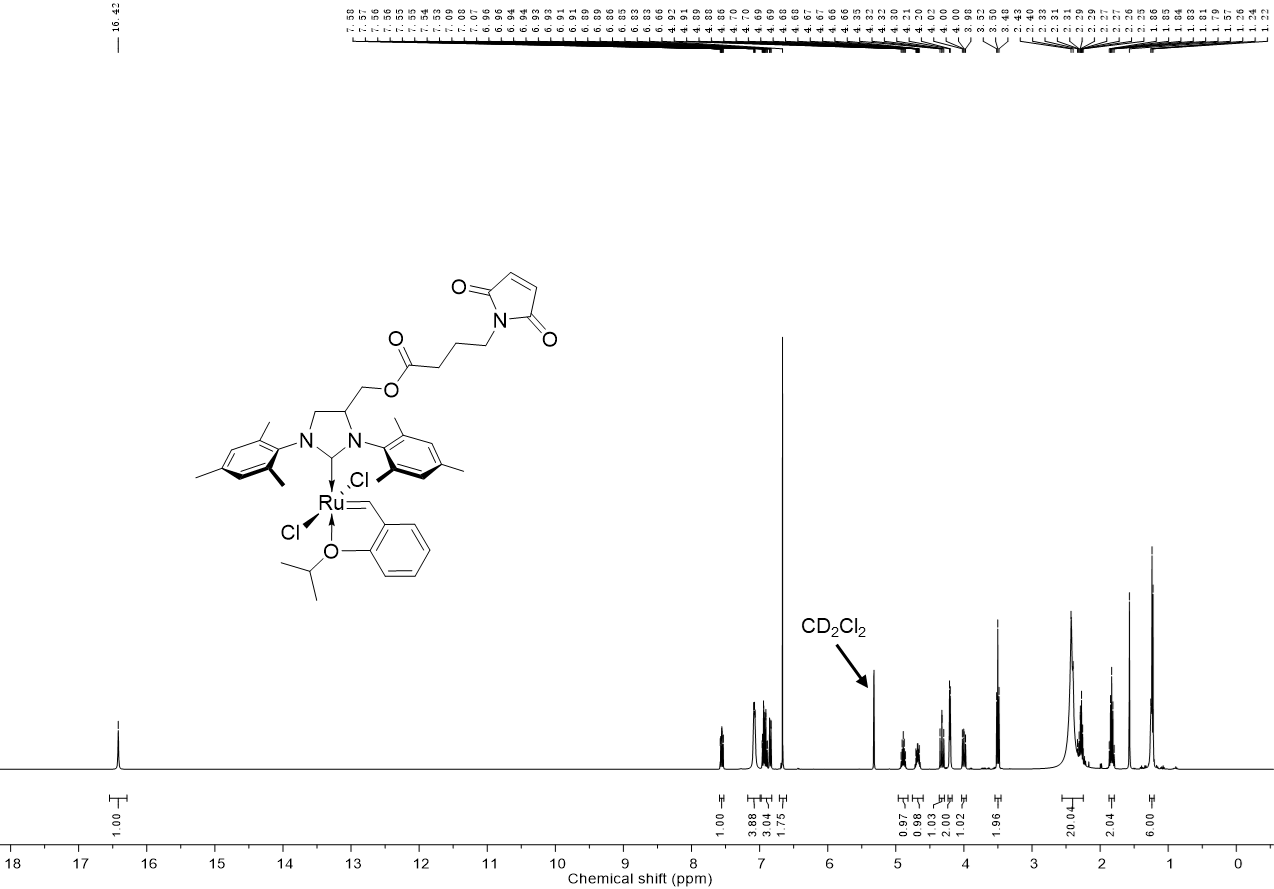


Figure S1. ^1^H NMR spectrum of GH-C3 (400 MHz, CD_2_Cl_2_, 298 K).


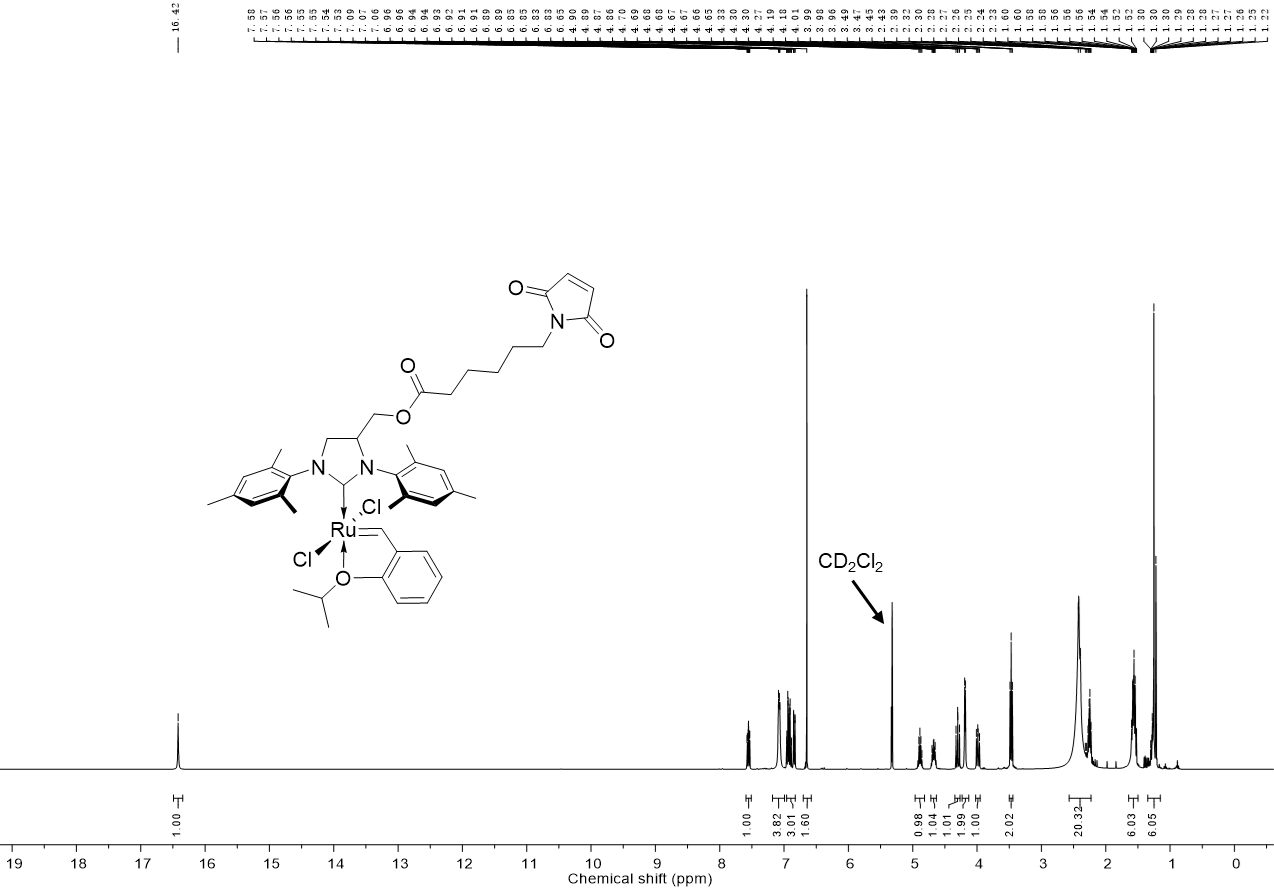


Figure S2. ^1^H NMR spectrum of GH-C5 (400 MHz, D_2_O, 298 K).


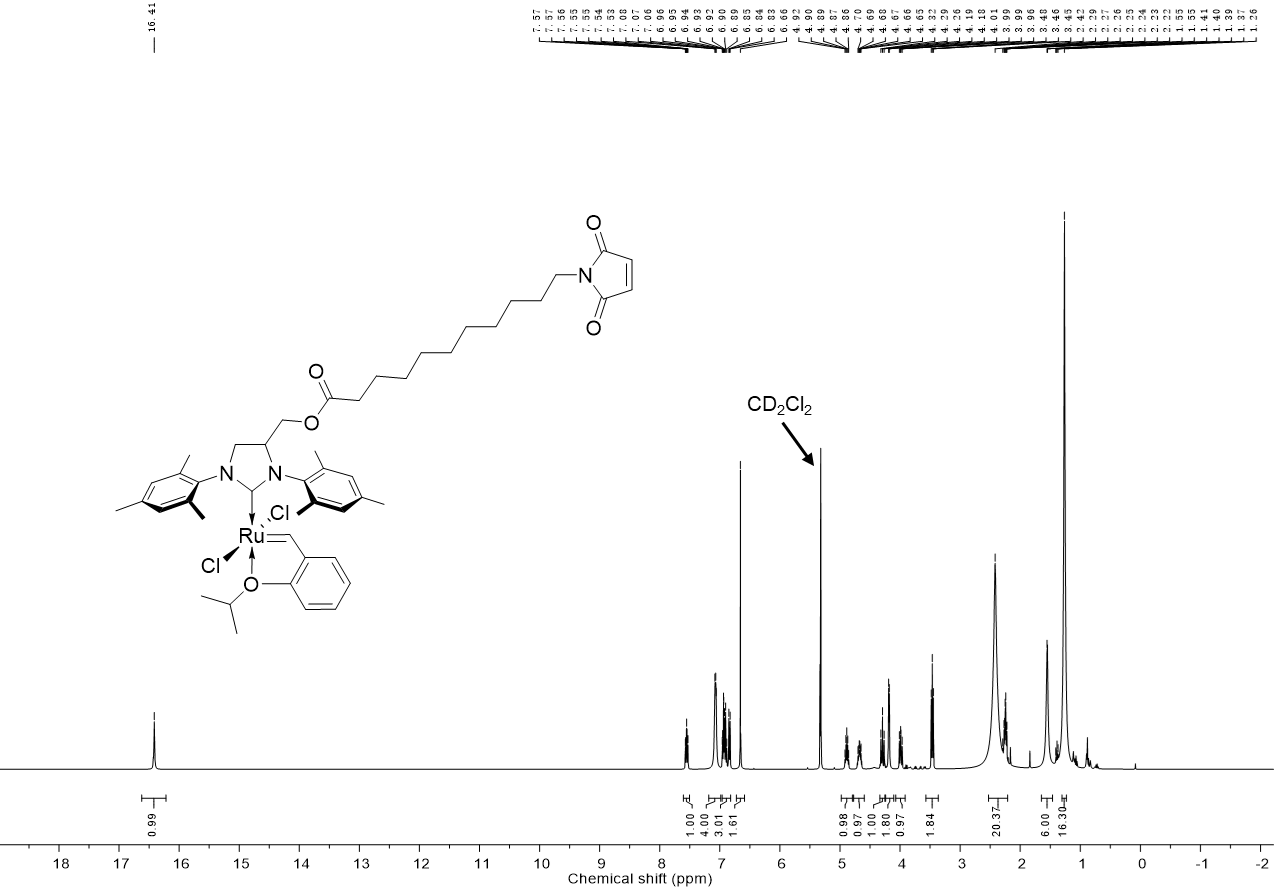


Figure S3. ^1^H NMR spectrum of GH-C10 (400 MHz, CD_2_Cl_2_, 298 K).


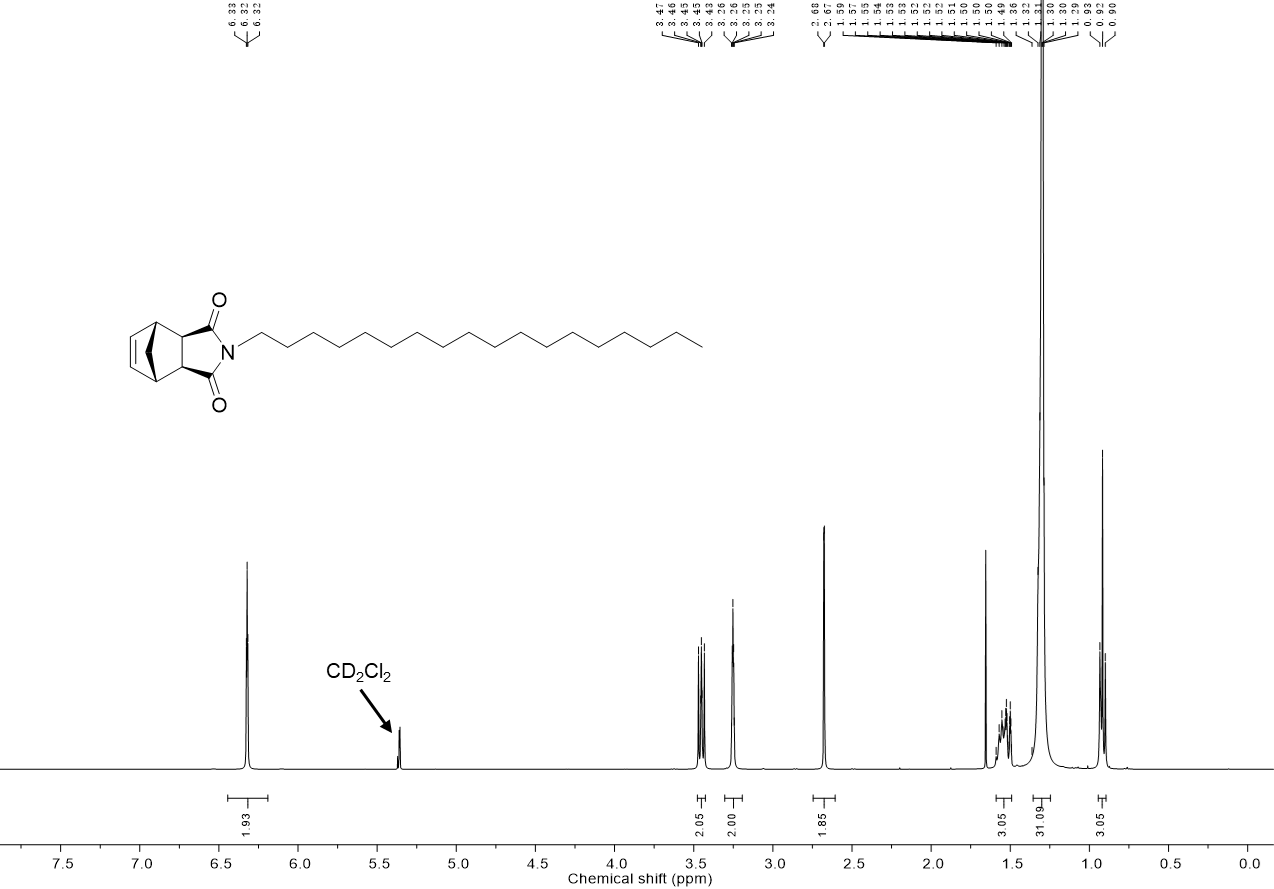


Figure S4. ^1^H NMR spectrum of Nor-C18 (400 MHz, CD_2_Cl_2_, 298 K).


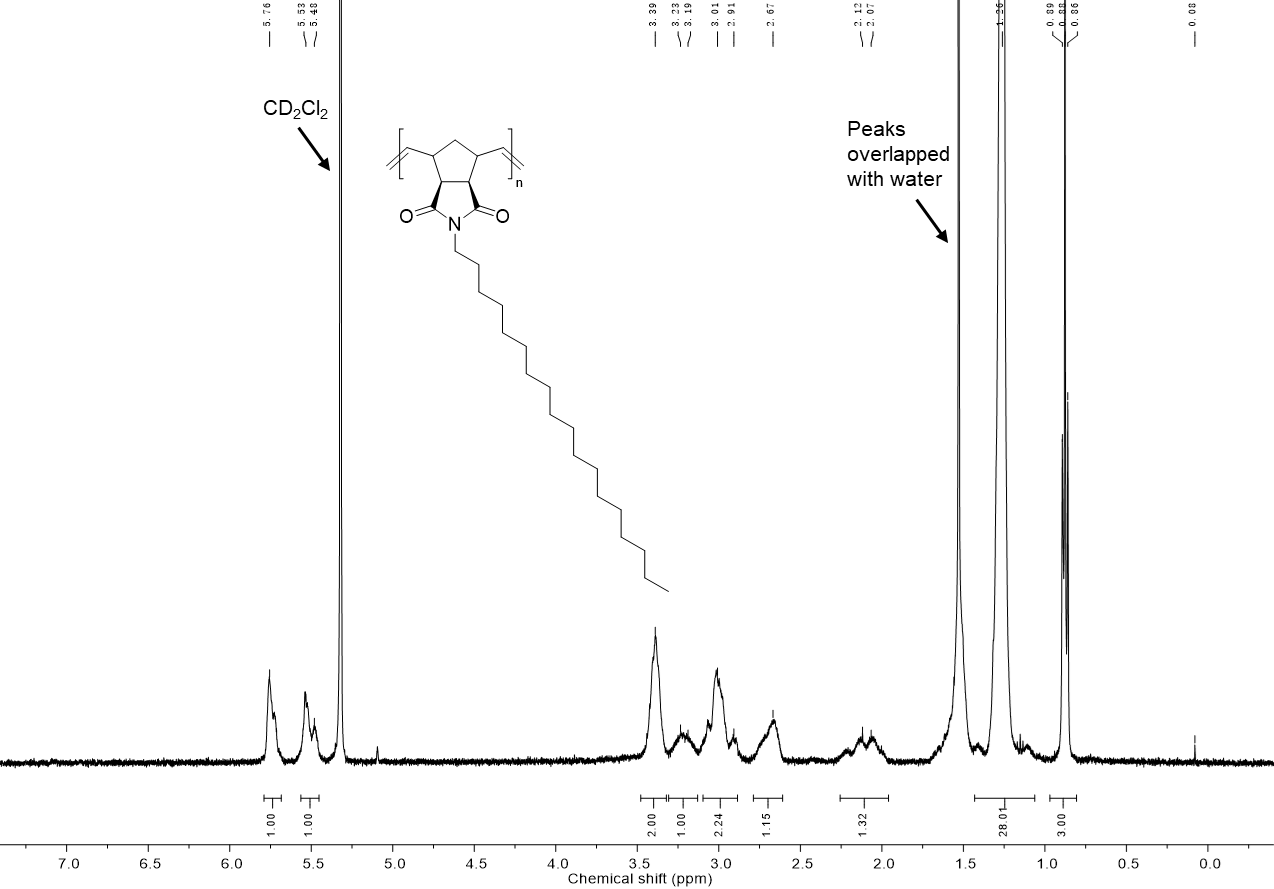


Figure S5. 1H NMR spectrum of the film formed after ROMP of Nor-C18 (400 MHz, CD2Cl2, 298 K).

# Supplementary Figures and Tables





Figure S6. Magnetization of bare Fe_3_O_4_ (blue) and film-coated Fe_3_O_4_ (red) through vibrating sample magnetometer.





Figure S7. Standard curve of LCI_F16C protein concentration. LCI_F16C with free thiol group can conjugate with ThioGlo-1 dye and show fluorescence. (y=10915.2x-191.2, R^2^=0.9994)





Figure S8. Standard curve of PS-COOH_500 nm_ at OD_460 nm_ measured by UV absorbance measurement (y=5.4455x+0.0041, R^2^=0.9998).





Figure S9. Recovery of bare and film-coated Fe_3_O_4_ (75 mg/L) for 0.1 g/L PS-COOH_500 nm_ NPs at 100 mM NaCl.

**Table S3**. Parameters of the adsorption kinetics equation of film-coated Fe_3_O_4_ against PS-COOH_500 nm_ NPs at different NaCl concentrations.

|  | Pseudo-first-order model | | | Pseudo-second-order model | | |
| --- | --- | --- | --- | --- | --- | --- |
| NaCl concentration | q_e_ (g/g) | k_1_ (min^-1^) | R^2^ | q_e_ (g/g) | k_2_ (g/(g·min)) | R^2^ |
| 100 mM | 1.845 ± 0.035 | 0.651 ± 0.046 | 0.992 | 2.103 ± 0.027 | 0.394 ± 0.025 | 0.998 |
| 150 mM | 1.898 ± 0.043 | 0.847 ± 0.079 | 0.986 | 2.121 ± 0.025 | 0.541 ± 0.035 | 0.998 |
| 200 mM | 1.986 ± 0.049 | 1.023 ± 0.111 | 0.980 | 2.187 ± 0.027 | 0.670 ± 0.050 | 0.997 |

**Table S4**. Parameters of the adsorption isotherm equation of film-coated Fe_3_O_4_ against PS-COOH_500 nm_ at different NaCl concentrations.

|  | Freundlich model | | | Langmuir model | | |
| --- | --- | --- | --- | --- | --- | --- |
| NaCl concentration | 1/n | K_F_ (L/g) | R^2^ | q_m_ (g/g) | K_L_ (L/g) | R^2^ |
| 100 mM | 0.218 ± 0.037 | 4.042 ± 0.084 | 0.924 | 4.602 ± 0.140 | 6.249 ± 0.140 | 0.999 |
| 150 mM | 0.274 ± 0.045 | 4.378 ± 0.110 | 0.928 | 5.209 ± 0.210 | 4.622 ± 0.686 | 0.999 |
| 200 mM | 0.278 ± 0.056 | 4.623 ± 0.146 | 0.894 | 5.529 ± 0.294 | 4.499 ± 0.867 | 0.998 |

Figure S10. SEM images of NPs investigated in this study, including PP, PS, PE, PET, PMMA, and SBR NPs.


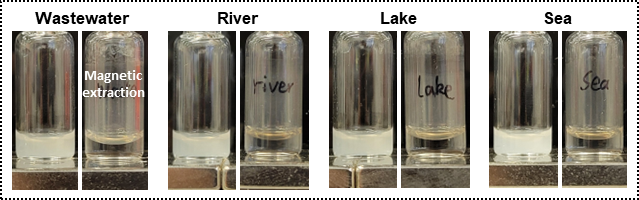


Figure S11. Visualization of 0.2 g/L of dispersed PS NPs in wastewater, river, lake, and sea before (left) and after magnetic extraction (right) by employing film-coated Fe_3_O_4_ beads.

# DNA Sequence of LCI_F16C

GCTGAATTCTCTGCATGGAGCCATCCGCAGTTCGAAAAGGCAGAAGCAGCAGCAAAAGAAGCCGCTGCCAAAGAAGCGGCAGCGAAAGCAGAAAATCTGTATTTTCAGGGGGCCATTAAACTGGTTCAGAGCCCGAATGGTAATTTTGCAGCAAGCTGTGTTCTGGATGGCACCAAATGGATCTTCAAAAGCAAATACTATGACAGCAGCAAAGGTTATTGGGTGGGTATTTATGAAGTGTGGGATCGCAAA

The mutation F16C is marked as red.

# References

[1] F. Philippart, M. Arlt, S. Gotzen, S.-J. Tenne, M. Bocola, H.-H. Chen, L. Zhu, U. Schwaneberg, J. Okuda, *Chem. Eur. J.* **2013**, *19*, 13865-13871.

[2] L. Ding, J. Qiu, Z. Zhu, *Macromol. Rapid Commun.* **2013**, *34*, 1635-1641.

[3] L. Chen, S. Li, Y. Ding, C. Wang, S. Zhang, R. Xu, Y. Chen, H. Li, M. Gao, Y. Qi, Y. Xu, X. Ma, L. Li, *ACS Med. Chem. Lett.* **2021**, *12*, 1589-1595.

[4] E. Krieger, G. Vriend, *Bioinformatics* **2014**, *30*, 2981-2982.

[5] Y. Duan, C. Wu, S. Chowdhury, M. C. Lee, G. Xiong, W. Zhang, R. Yang, P. Cieplak, R. Luo, T. Lee, J. Caldwell, J. Wang, P. Kollman, *J. Comput. Chem.* **2003**, *24*, 1999-2012.

[6] A. Onoda, K. Fukumoto, M. Arlt, M. Bocola, U. Schwaneberg, T. Hayashi, *Chem. Commun.* **2012**, *48*, 9756-9758.

[7] K. Fukumoto, A. Onoda, E. Mizohata, M. Bocola, T. Inoue, U. Schwaneberg, T. Hayashi, *ChemCatChem* **2014**, *6*, 1229-1235.

[8] J. Wang, R. M. Wolf, J. W. Caldwell, P. A. Kollman, D. A. Case, *J. Comput. Chem.* **2004**, *25*, 1157-1174.

[9] A. Jakalian, D. B. Jack, C. I. Bayly, *J. Comput. Chem.* **2002**, *23*, 1623-1641.

[10] S. B. Garber, J. S. Kingsbury, B. L. Gray, A. H. Hoveyda, *J. Am. Chem. Soc.* **2000**, *122*, 8168-8179.

[11] J. Wang, X. Guo, *J. Hazard. Mater.* **2020**, *390*, 122156.

[12] M. A. Al-Ghouti, D. A. Da'ana, *J. Hazard. Mater.* **2020**, *393*, 122383.

[13] F. H. Zokaei, S. Gharavi, E. Asgarani, M. Zarrabi, M. Soudi, Z. Moosavi-nejad, *Biologia* **2023**, *78*, 179-186.

[14] K. Vogel, R. Wei, L. Pfaff, D. Breite, H. Al-Fathi, C. Ortmann, I. Estrela-Lopis, T. Venus, A. Schulze, H. Harms, U. T. Bornscheuer, T. Maskow, *Sci. Total Environ.* **2021**, *773*, 145111.
